# Protein and DNA Biosynthesis Demonstrated in Host Cell-Free Phagosomes Containing Anaplasma phagocytophilum or Ehrlichia chaffeensis in Axenic Media

**DOI:** 10.1128/IAI.00638-20

**Published:** 2021-03-17

**Authors:** Yuntao Zhang, Li Chen, Chandramouli Kondethimmanahalli, Huitao Liu, Roman R. Ganta

**Affiliations:** a Center of Excellence for Vector-Borne Diseases, Department of Diagnostic Medicine/Pathobiology, College of Veterinary Medicine, Kansas State University, Manhattan, Kansas, USA; Yale University School of Medicine

**Keywords:** axenic medium, cell-free culture, phagosomes, *A. phagocytophilum*, *E. chaffeensis*, *Anaplasma*, *Ehrlichia*, host cell-free culture, obligate, rickettsiae, tick-borne pathogens

## Abstract

Rickettsiae belong to the *Anaplasmataceae* family, which includes mostly tick-transmitted pathogens causing human, canine, and ruminant diseases. Biochemical characterization of the pathogens remains a major challenge because of their obligate parasitism. We investigated the use of an axenic medium for growth of two important pathogens—Anaplasma phagocytophilum and Ehrlichia chaffeensis—in host cell-free phagosomes. We recently reported that the axenic medium promotes protein and DNA biosynthesis in host cell-free replicating form of *E. chaffeensis*, although the bacterial replication is limited. We now tested the hypothesis that growth on axenic medium can be improved if host cell-free rickettsia-containing phagosomes are used. Purification of phagosomes from A. phagocytophilum- and *E. chaffeensis*-infected host cells was accomplished by density gradient centrifugation combined with magnet-assisted cell sorting. Protein and DNA synthesis was observed for both organisms in cell-free phagosomes with glucose-6-phosphate and/or ATP. The levels of protein and DNA synthesis were the highest for a medium pH of 7. The data demonstrate bacterial DNA and protein synthesis for the first time in host cell-free phagosomes for two rickettsial pathogens. The host cell support-free axenic growth of obligate pathogenic rickettsiae will be critical in advancing research goals in many important tick-borne diseases impacting human and animal health.

## Abstract (precis)

Rickettsiae belong to the *Anaplasmataceae* family, which includes mostly tick-transmitted pathogens causing human, canine, and ruminant diseases. Biochemical characterization of the pathogens remains a major challenge because of their obligate parasitism.

## INTRODUCTION

Members of the *Anaplasmataceae* family, including Anaplasma phagocytophilum and Ehrlichia chaffeensis, are obligate, Gram-negative, intracellular rickettsiae responsible for causing in humans the acute febrile illnesses human granulocytic anaplasmosis (HGA) and human monocytic ehrlichiosis (HME) (1), respectively. These pathogens also cause infections in several vertebrate hosts (1). These two pathogens are responsible for the second most commonly reported tick-borne illnesses in the United States, causing significant morbidity and mortality. The diseases are also frequently reported in parts of Europe and Asia (1). The diseases can be fatal, particularly in immunocompromised individuals, the elderly, and children (2, 3). People undergoing blood transfusions and organ transplantations are also at high risk in acquiring the diseases (4–6).

A. phagocytophilum survives and propagates within the granulocyte phagosomes by evading neutrophil antimicrobial functions (7, 8), whereas *E. chaffeensis* replicates similarly in phagosomes of monocytes and macrophages (9, 10). The life cycles of A. phagocytophilum and *E. chaffeensis* involve a tick vector and a mammalian host. Both pathogens undergo transition between smaller cells with an electron-dense core (DCs), having a dense nucleoid, and cells with a larger, pleomorphic electron lucent form known as reticulate cells (RCs), which have a dispersed nucleoid (11, 12). The DC is the infectious form, while the RC is noninfectious and replicates within a phagosome. Between 4 and 12 h, a phagocytized DC form transforms to the RC form and replicates within a phagosome until reverting to the DC form and undergoing subsequent release from the infected host cells by completing cell lysis or exocytosis (11, 12).

The ability to grow obligate intracellular bacteria under axenic conditions can be a major advancement (13–15), as it will enable new paths of investigation, such as aiding the manipulation of the pathogenic organisms in the absence of host cells, clonal purification of bacterial mutants, and allowing detailed biochemical and genetic studies. The development of axenic medium for growth and its application are well documented in another important obligate bacterium, Coxiella burnetii, and the method aided greatly in studies focused on biochemical and genetic studies of the pathogen (16–18). While such efforts have been attempted for another important bacterial pathogen, Chlamydia trachomatis, only limited protein synthesis was reported (19). Similarly, we recently assessed the application of axenic media for *E. chaffeensis*, where we demonstrated both protein and DNA biosynthesis for the RC form of the pathogen (20). As protein synthesis and DNA replication are limited in the axenic media, we proposed several strategies to promote optimal cell-free bacterial replication (20). Furthermore, such methods to grow *Anaplasma* species pathogens in host cell-free media have yet to be developed.

Considering the potential advancements likely achieved by growth on axenic media of the pathogenic rickettsiae, we extended investigations in this follow-up study to improve the axenic growth conditions for *E. chaffeensis* and also initiated similar experiments for A. phagocytophilum. In this study, we present novel data demonstrating the purification of host cell-free phagosomes containing A. phagocytophilum or *E. chaffeensis* and then used the phagosomes to assess the bacterial protein and DNA synthesis under axenic medium conditions.

## RESULTS

### Rickettsia-containing phagosome purification and verification.

**FIG 1:**
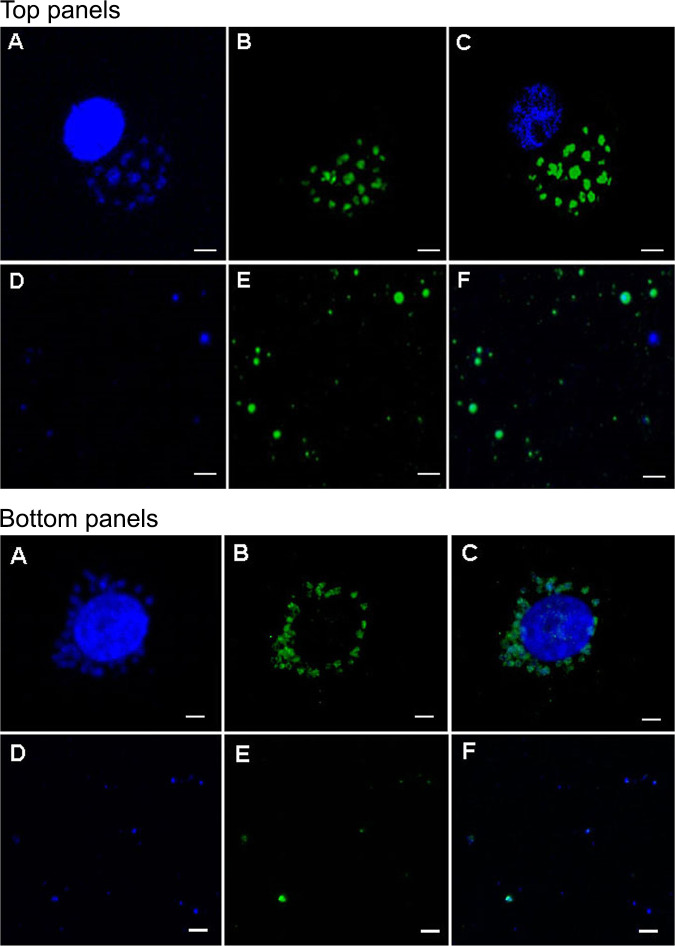
(Top panels A to F) Representative confocal images showing phagosomes of the HL-60 cells infected by A. phagocytophilum stained with DAPI (A) and Rab 5 monoclonal antibody (B). A merged image is shown in panel C. Purified A. phagocytophilum phagosomes recovered from infected HL-60 cells were similarly stained with DAPI and Rab 5 antibody (panels D and E, respectively, with panel F representing the merged image). Scale bars in each panel are 5 μm. Bottom panels A to F present the data for *E. chaffeensis* cultured in DH82 cells. The image descriptions for bottom panels A to F are identical to descriptions for the top panels, including the scale bar size.

We adopted the ultracentrifugation method using discontinuous sucrose density gradient coupled with magnet-assisted cell sorting (MACS) to purify phagosomes from A. phagocytophilum-infected HL-60 cells and similarly *E. chaffeensis*-infected DH82 cells (Fig. 1). Confocal microscopy evaluation following staining with DAPI (4′,6-diamidino-2-phenylindole) for nuclear material and phagosome membrane-specific Rab 5 monoclonal antibody, respectively, were used to confirm the purity of phagosomes containing the rickettsial organisms.

### Assessment of protein synthesis in cell-free phagosomes in axenic media.

**FIG 2:**
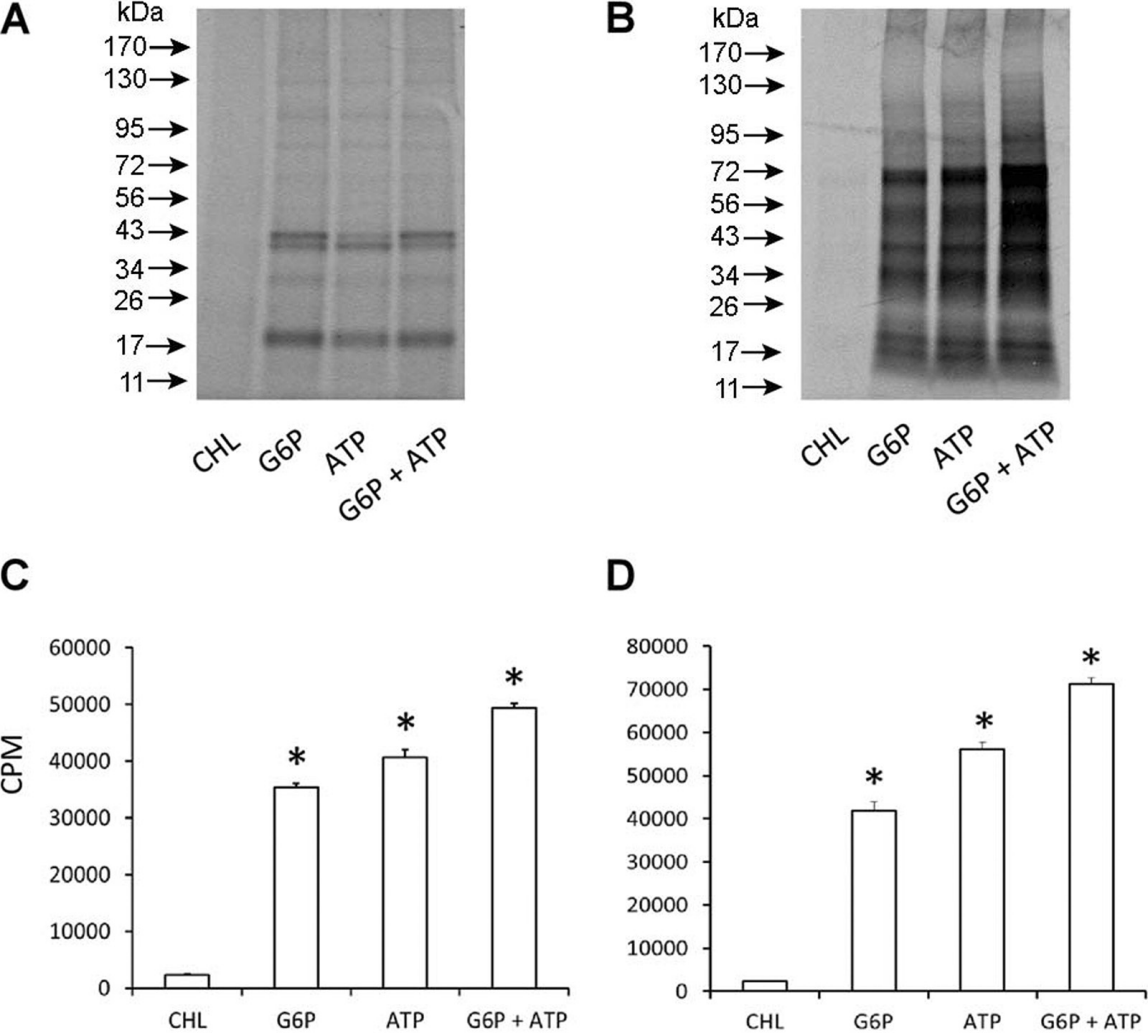
Impact of energy sources on incorporation of [^35^S]Cys-Met into phagosomes. (A and C) *Anaplasma* cell-free phagosome. (B and D) *E. chaffeensis* cell-free phagosome. The first lane included axenic media with G6P, ATP, and chloramphenicol (CHL) to serve as the negative control. The [^35^S]Cys-Met incorporation was assessed for 24 h at 37°C with 2.5% O_2_. The pH of the negative-control medium was adjusted to 7.

Previously described axenic medium (20, 21) was used with two different carbon/energy sources—glucose-6-phosphate (G6P) or ATP—or by adding both metabolites to assess protein synthesis in cell-free phagosomes containing either A. phagocytophilum or *E. chaffeensis*. Rickettsiae containing cell-free phagosomes were incubated with the axenic media for 24 h at 37°C in a tri-gas incubator set to maintain 2.5% O_2_. The concentration of Cys and Met in the axenic media was reduced to 1 μM, and then the two radioactive amino acids were supplemented with 70 μCi of [^35^S]Cys-Met. Protein synthesis was assessed by monitoring the [^35^S]Cys-Met incorporation. Phagosomes isolated from the A. phagocytophilum-infected host cells and similarly from *E. chaffeensis*-containing purified phagosomes utilized either G6P or ATP, as judged by the incorporation of [^35^S]Cys-Met (Fig. 2). The protein synthesis was not significantly different for G6P or ATP alone, although the addition of both resulted in notable increase. The incorporation of radioactive amino acids was completely absent when chloramphenicol was added to the axenic medium to arrest protein synthesis.

### Impact of pH on protein biosynthesis in cell-free phagosomes in the axenic media.

**FIG 3:**
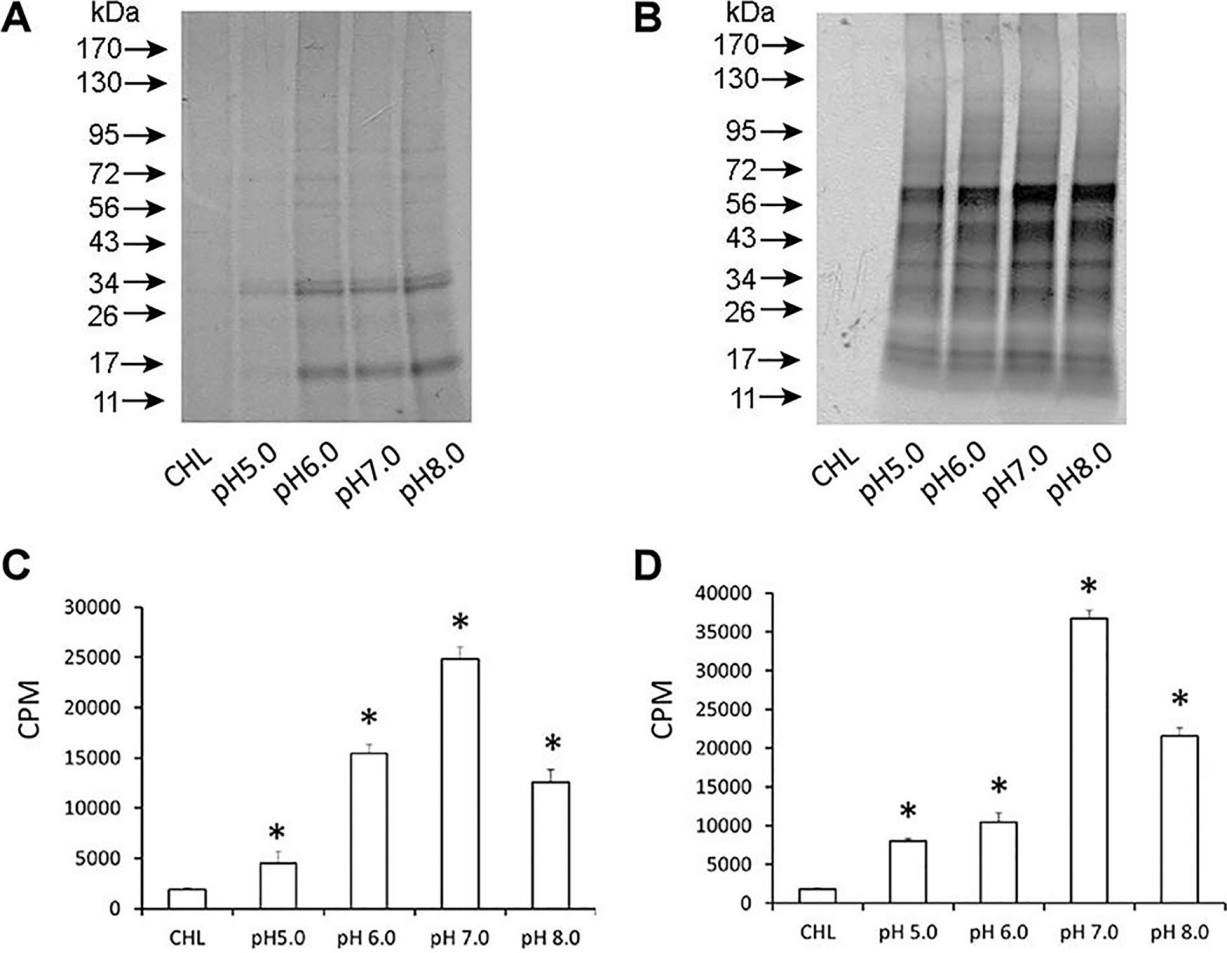
Impact of pH variations assessed on the [^35^S]Cys-Met incorporation into phagosome. (A and C) *Anaplasma* cell-free phagosome. (B and D) *E. chaffeensis* cell-free phagosome. The first lane included axenic media with G6P, ATP, and chloramphenicol (CHL) to serve as the negative control. The pH of the negative-control medium was adjusted to 7. The incubation time and conditions were as in Fig. 2.

We assessed the pH variations in promoting optimal protein biosynthesis in purified phagosomes containing either A. phagocytophilum or *E. chaffeensis* (Fig. 3). Although the [^35^S]Cys-Met incorporation was observed in the axenic media at pH variations from 5 to 8, the highest incorporation was observed for the media at pH 7 for both *E. chaffeensis*- and A. phagocytophilum-containing phagosomes.

### Restricted protein biosynthesis observed when total protein synthesis was assessed by polyacrylamide gel electrophoresis and by Western blotting.

**FIG 4:**
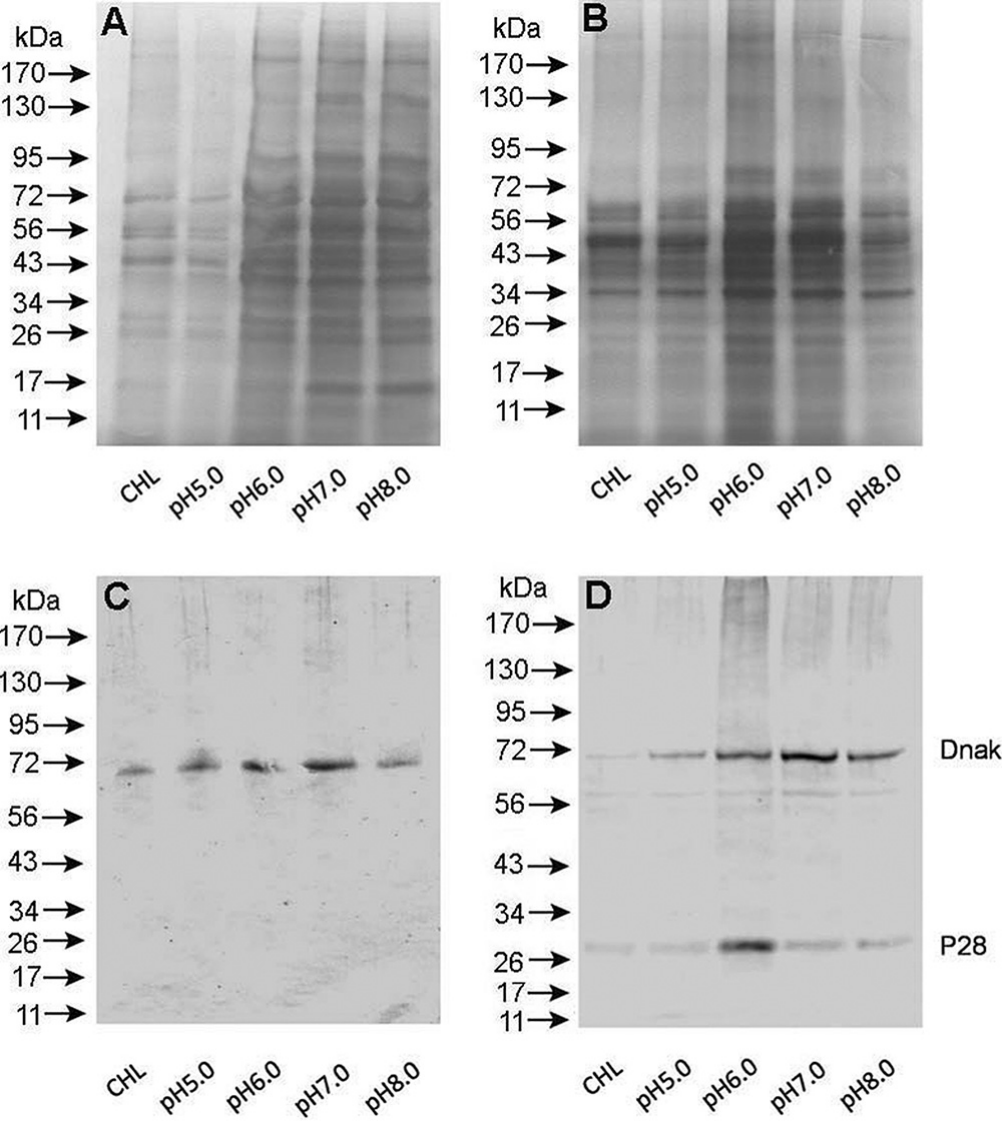
Protein biosynthesis assessed by protein fractionation and Western blot analysis. (A) Silver-stained SDS-containing polyacrylamide gel-resolved protein fractions were assessed for protein abundance variations in *Anaplasma* cell-free phagosomes. (B) Silver-stained SDS-containing polyacrylamide gel-resolved protein fractions were assessed for protein abundance variations in *E. chaffeensis* cell-free phagosomes. (C) As in panel A, protein biosynthesis was assessed by Western blotting using rabbit polyclonal sera against recombinant *E. chaffeensis* DnaK and mouse monoclonal antibody against P28-OMP19. (D) As in panel B, protein biosynthesis was assessed by Western blotting using the antibodies listed above.

We then assessed the level of protein biosynthesis by measuring protein levels relative to controls for both of the rickettsial organisms in host cell-free phagosomes maintained in the axenic media. Total protein profiles of the resolved proteins in a polyacrylamide gel following silver staining showed an increase in total proteins compared to the control reaction mixtures in which chloramphenicol was added (Fig. 4A and B). However, the level of synthesized proteins appeared only moderately higher than that of the controls, as assessed for 24 h of incubation in the axenic media. Maximum total protein synthesis was observed for both A. phagocytophilum and *E. chaffeensis* phagosomes when the pH of the medium was adjusted to 7. These data were further confirmed following Western blot analysis (Fig. 4C and D). Two different antibodies were used for this experiment: polyclonal sera raised against *E. chaffeensis* DnaK protein and a monoclonal antibody for the organism’s outer membrane protein, P28-OMP19. The DnaK antibody detected a protein band of the expected size in *E. chaffeensis*, and this antibody also recognized a protein of similar size in A. phagocytophilum phagosome lysate, while the P28-OMP19 monoclonal antibody recognized only *E. chaffeensis* protein. Independent of the variations in the pH of the axenic media, the increase in total protein synthesis was only moderate compared to the greatest difference in synthesis noted for the reaction for the media at pH 7 compared to the controls containing chloramphenicol.

### DNA synthesis assessed in axenic media is analogous to protein biosynthesis.

**FIG 5:**
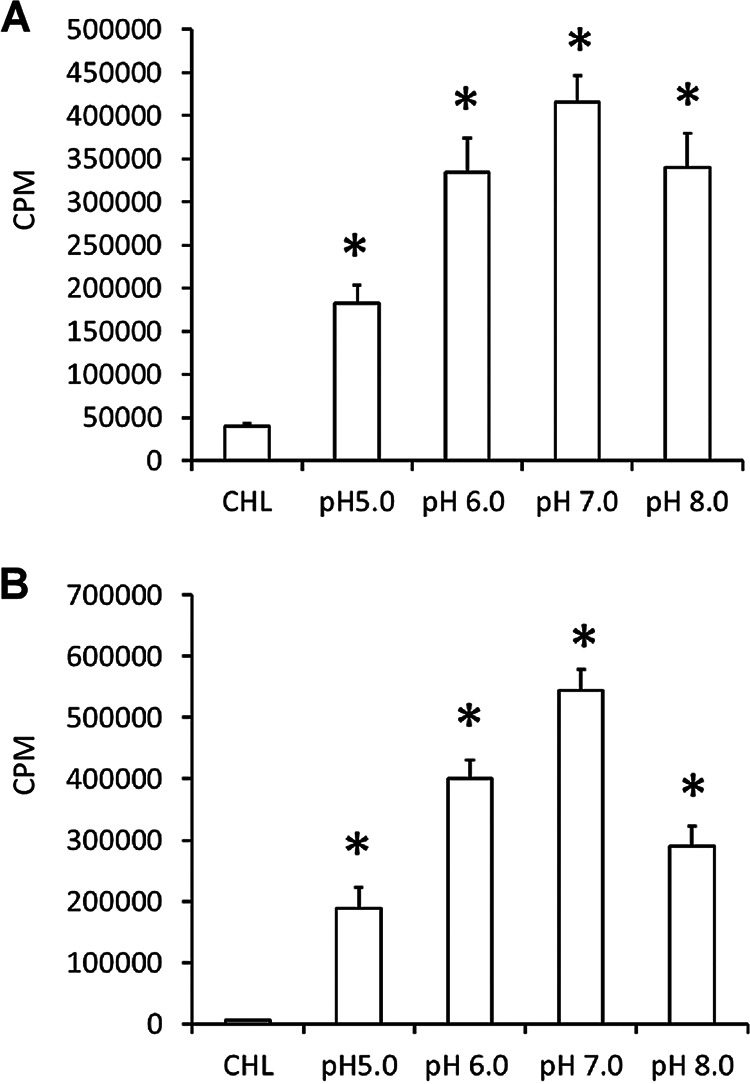
DNA synthesis assessed simultaneously by measuring the [^3^H]thymidine incorporation in the axenic media at various pHs of the media. (A) *Anaplasma* cell-free phagosome. (B) *E. chaffeensis* cell-free phagosome. The first column included axenic media with G6P, ATP, and chloramphenicol (CHL) to serve as the negative control. The pH of the negative-control medium was adjusted to 7. The incubation times and conditions were similar to those described in the legend to Fig. 2.

To determine if the axenic media also promoted DNA synthesis, rickettsia-containing phagosomes were incubated in the media containing [^3^H]thymidine (Fig. 5). This experiment was also performed at variant pHs of the media. DNA synthesis was observed in the axenic media for purified phagosomes containing *E. chaffeensis* or A. phagocytophilum. Consistent with the [^35^S]Cys-Met incorporation, the increase in DNA synthesis in the cell-free phagosomes incubated in the axenic media was observed, while maximum incorporation was detected for the media at pH 7.

### RNA synthesis assessed in axenic media was also consistent with DNA and protein biosynthesis.

We performed a quantitative reverse transcription-PCR (qRT-PCR) analysis targeting 16S rRNA for the purified phagosomes containing A. phagocytophilum or *E. chaffeensis*. The analysis was performed on RNA recovered at different hours of incubation in the axenic media for 0, 2, 6, 12, and 24 h. RNA samples in triplicate reactions were assessed for each incubation time point by TaqMan probe-based, real-time qRT-PCR targeting the bacterial 16S rRNA (Table 1). There was no significant change in the RNA expression levels between 0 h and 2 h of incubation for *E. chaffeensis*, while RNA copy numbers beyond 2 h for *E. chaffeensis* and for A. phagocytophilum starting at 2 h of incubation declined rapidly, possibly due to the rapid loss of viability of the cell-free rickettsia-containing phagosomes in the media after the first 2 h of incubation, where protein biosynthesis and DNA and RNA synthesis may have occurred.

**TABLE 1:** *E. chaffeensis* and A. phagocytophilum 16S rRNA assessed by TaqMan probe-based real-time qRT-PCR assays for RNA^*a*^

Incubation period (h)	*C_T_* (SD)	Fold reduction compared to 0 h	*P* value^*b*^
*E. chaffeensis*			
0	14.89 (0.24)	NA^*c*^	NA
2	15.96 (0.80)	2	0.0811
6	17.93 (0.26)	8	0.0056*
12	21.65 (0.23)	108	0.0016*
24	26.79 (0.31)	3,821	0.0001*

A. phagocytophilum			
0	7.34 (0.81)	NA	NA
2	13.98 (2.22)	99	0.0329*
6	26.76 (1.01)	701,459	0.0459*
12	27.49 (1.50)	1,163,467	0.0344*
24	30.99 (0.07)	13,163,136	0.0170*

a 16S rRNA was assessed by TaqMan probe-based real-time qRT-PCR assays for RNA recovered from purified phagosomes following the different time points shown of incubation in the axenic media at pH 7.

b Asterisks indicate significant fold change at different time points compared to the 0-h value.

c NA, not applicable.

## DISCUSSION

The lack of methods to grow most of the obligate pathogens, including rickettsial pathogens, in the absence of host cells remains a major limiting factor hampering research progress (19, 20). The importance of the application of the axenic medium method to grow an obligate pathogenic bacterium is well recognized from the rapid advances made following its development for Coxiella burnetii (14, 16–18, 22). In an effort to develop such methods for rickettsial pathogens, we recently described the first evidence of application of axenic growth medium for *E. chaffeensis* (20). In that study, we demonstrated protein and DNA synthesis only in the RC form, but not in the DC form, in axenic medium formulations for host cell-free *E. chaffeensis*. We recognized that additional investigations are necessary to improve the experimental conditions optimal for promoting the continued replication of *E. chaffeensis* organisms (20). These observations are similar to a prior study demonstrating cell-free protein biosynthesis for C. trachomatis (19). Furthermore, cell-free medium growth studies have not been investigated to date for any *Anaplasma* species pathogens. We reasoned that the axenic medium growth of the cell-free RC form of *E. chaffeensis* may be improved if assessed with purified, host cell culture-derived *E. chaffeensis* in phagosomes in place of purified RCs. The phagosome microenvironment might mimic *in vivo* conditions. In the current study, therefore, we investigated this goal for *E. chaffeensis*. The investigations were also extended to A. phagocytophilum as there was no prior research focused on development of axenic media for any known *Anaplasma* species pathogens. *Anaplasma* species pathogens resulting from tick transmission cause significant morbidity and mortality in various agricultural and companion animals, besides impacting human health (23). Similarly, infections with several tick-borne *Ehrlichia* species pathogens impact the health of companion and agricultural animals and people (1, 24). The availability of axenic medium culture systems for *Anaplasma* and *Ehrlichia* species pathogens, therefore, will greatly aid in advancing our understanding of these important microorganisms.

We first standardized the method to purify rickettsia-containing phagosomes and then utilized the purified phagosomes to investigate protein and DNA synthesis with axenic media under microaerophilic conditions. Similar to our prior observations for the host cell-free RC form of *E. chaffeensis*, the current study demonstrated protein biosynthesis and DNA synthesis in the axenic media for both *E. chaffeensis*- and A. phagocytophilum-containing phagosomes. Axenic medium-specific protein biosynthesis and DNA synthesis were confirmed for purified phagosomes with the inclusion of chloramphenicol in the media to serve as the protein synthesis inhibitor. The rickettsial protein and DNA biosynthesis was reported for the first time for phagosomes *in vitro* in the current study. While it is significant to demonstrate the data for host cell-free phagosomes containing rickettsial organisms, there was considerably less protein and DNA biosynthesis. These data are similar to our prior observations for the purified RC form of *E. chaffeensis* (20). The assessment of total proteins synthesized in axenic media for phagosomes, as judged from the total protein analysis of silver-stained gels of resolved proteins and by Western blotting, suggested only a moderate enhancement of protein synthesis for both A. phagocytophilum and *E. chaffeensis*. These data are similar to our earlier reported axenic medium data for the host cell-free *E. chaffeensis* RCs (20). Notably, contrary to our predictions, axenic media promoted only limited protein and DNA synthesis for *Ehrlichia*- or *Anaplasma*-containing phagosomes. Albeit the current study is a major step forward for improving growth on axenic media because this is the first study examining the utility of purified phagosomes containing two important rickettsial pathogens belonging to members of the *Anaplasmataceae* family, additional modifications are required to stimulate greater DNA and protein biosynthesis.

We recognized that the *in vitro*-purified *E. chaffeensis*- and A. phagocytophilum-containing phagosomes rapidly lost their viability in the axenic medium culture conditions, as judged from the rapid loss of RNA copy number after about 2 h of incubation under the axenic medium conditions. The reasons for the rapid decline in viability are unclear. One possibility could be the extended time involved in the phagosome purification procedure. Alternatively, the axenic medium conditions may require further optimization. Importantly, it is evident that improvements should be made to sustain the viability during the axenic medium incubation time. We are currently investigating ways to reduce the purification time. Additional advances are also necessary to maintain the integrity of the recovered rickettsia-containing phagosomes, which may include the choice of harvest time following *in vitro* culture setup, as well as the host cell selection for propagation of A. phagocytophilum and *E. chaffeensis*, all of which may contribute to the stability of phagosomes and the promotion of growth on axenic media.

While the purification protocol is more laborious and time-consuming than purifying the RC form of bacteria, improvements may be possible to promote the bacterial growth in cell-free rickettsia-containing phagosomes. Transmission electron microscopy studies by us and other investigators demonstrated that mitochondria are closely associated with *E. chaffeensis*-containing phagosome vacuoles of infected host cells (11, 25, 26). *E. chaffeensis* and other *Anaplasmataceae* pathogens may benefit from mitochondria in multiple ways, including obtaining energy and metabolites. Axenic medium growth of A. phagocytophilum and *E. chaffeensis* in phagosomes recovered from *in vitro* cultures, therefore, may also be improved by the addition of purified mitochondria to the axenic media. Likewise, axenic medium growth for the RC forms of both *Anaplasma* and *Ehrlichia* species may be perfected in ways to promote bacterial synthesis *in vitro*. We believe that the complementary approaches using host cell-free rickettsia-containing phagosomes and RC forms may need to be simultaneously investigated to advance the goal of developing optimal protocols for axenic growth of *Anaplasmataceae* pathogens.

The current study describing the established methods of phagosome purification and their use in promoting protein and DNA biosynthesis *in vitro* under axenic medium culture conditions forms a strong foundation for making further improvements to promote sustained and enhanced replication of pathogenic rickettsiae in the absence of host cell support. Future investigations may also be extended to important tick-borne obligate rickettsial pathogens of the genera *Anaplasma*, *Ehrlichia*, and *Neorickettsia* to promote axenic medium growth to induce rapid bacterial replication and progression from the RC form to DC form. The host cell support-free axenic growth of obligate *Anaplasmataceae* pathogens will be critical in advancing research goals in many important tick-borne diseases impacting human and animal health.

## MATERIALS AND METHODS

### Cell lines and cultivation of A. phagocytophilum and *E. chaffeensis*.

Cells of the human promyelocytic cell line HL-60 (ATCC CCL-240; ATCC, Manassas, VA), uninfected or infected with A. phagocytophilum strain NCH-1, were cultured in complete RPMI 1640 medium (Gibco/Thermo Fisher Scientific) supplemented with 10% fetal bovine serum (Invitrogen/Thermo Fisher Scientific, Waltham, MA) and 2 mM l-glutamine (Mediatech, Manassas, VA) by following the protocols described in reference 7. Cultivation of *E. chaffeensis* in DH82 cells was performed in complete RPMI 1640 medium (Gibco/Thermo Fisher Scientific) supplemented with 10% fetal bovine serum (Invitrogen/Thermo Fisher Scientific) and 2 mM l-glutamine (Mediatech) as per protocols described previously (20). To prepare cell-free inocula, about 80 to 100% A. phagocytophilum-infected HL-60 cells or *E. chaffeensis*-infected DH82 cells from T25 flasks were harvested by centrifugation at 400 × *g* for 10 min at 4°C. The pellets were resuspended in 5 ml of serum-free medium, and the cells were disrupted with glass beads by vortexing twice for 30 s. The cell debris and unbroken cells were removed by centrifugation at 200 × *g* for 10 min at 4°C. The supernatant was passed through a 2.7-μm-pore syringe filter (Whatman, Pittsburgh, PA). HL-60 and DH82 cells were infected with host cell-free A. phagocytophilum and *E. chaffeensis*, respectively (at a multiplicity of infection of 100:1 bacteria to host cells) for 2 h to allow internalization. Noningested rickettsial organisms were removed by washing with phosphate-buffered saline (PBS), and the cells were incubated for 36 to 48 h in a T150 flask. The infected host cell cultures were harvested by centrifugation at 600 × *g* for 5 min at 4°C and used for purification of the host cell-free phagosomes, as outlined below.

### Purification of phagosomes.

Purification of phagosomes from the A. phagocytophilum-infected HL-60 cells and *E. chaffeensis*-infected DH82 cells was performed by subjecting them to discontinuous sucrose density gradient centrifugation (27) in combination with magnet-assisted cell sorting (MACS) as described previously (28, 29), with some minor modifications. In brief, infected host cells were pelleted at 4°C for 5 min at 350 × *g*. The cells were washed twice with PBS and once with homogenization buffer (250 mM sucrose, 0.5 mM EGTA, and 20 mM HEPES-KOH at pH 7.2). The cells were then resuspended in homogenization buffer with protease inhibitor cocktail (Sigma-Aldrich, St. Louis, MO). The cells were then homogenized at 4°C using a 10-ml syringe with a 23.5-gauge needle; typically, 10 to 15 strokes were used to disrupt the cells. Homogenization was carried out until approximately 90% of cells were disrupted without major breakage of nuclei, as monitored by light microscopy. The unlysed host cells and nuclei were then pelleted in a 15-ml tube centrifuged at 300 × *g* at 4°C for 5 min. The resulting supernatant containing phagosomes, which was designated the postnuclear supernatant (PNS), was brought to a final concentration of 39% sucrose. The discontinuous sucrose gradient was made by layering 1 ml of PNS in 39% sucrose onto 2 ml of 55% sucrose layered onto 1 ml of 65% sucrose. We then layered 2 ml of 25% sucrose followed by 2 ml of 10% sucrose solution. The gradient was centrifuged at 100,000 × *g* for 1 h at 4°C with an S50-ST swinging bucket rotor in a Sorvall MTX150 ultracentrifuge (Waltham, MA). Phagosomes were then recovered from the interface between 55% and 65% sucrose using a 16-gauge needle carefully without disrupting other gradient fractions. Subsequently, the MACS separation step was performed by incubation of the crude phagosomes with rabbit Rab5 antibody (1:1,000; Cell Signaling Technology, Danvers, MA) for 1.5 h, followed by incubation with a secondary goat anti-rabbit antibody with magnetic beads (1:100; Miltenyi Biotec, San Diego, CA) for another 1.5 h, and then loaded onto a MACS LS separation column (Miltenyi Biotec, San Diego, CA). The column was washed three times with 2 ml HSMG (20 mM HEPES, 250 mM sucrose, 1.5 mM MgCl_2_, 0.5 mM EGTA at pH 7.4), and on removal of the magnet, the phagosomes were eluted in 3 ml HSMG. The elution product was placed into 10 ml of PBS (4°C) and centrifuged at 40,000 × *g* for 30 min at 4°C with an S50-ST swinging bucket rotor in a Sorvall MTX150 ultracentrifuge. The final purified pellets were resuspended in PBS for immediate use in the cell-free axenic medium assessment experiments.

### Confocal microscopy analysis.

A fraction of purified phagosomes was plated onto 8-well culture chamber slide to adhere for 1 h, and then stained with rabbit Rab5 (1:1000) (Cell Signaling Technology, Danvers, MA) antibody for 1.5 h and with Alexa Fluor 488-conjugated goat anti-rabbit antibody for 1 h. The slides were washed with PBS and mounted with mounting medium containing DAPI. For staining of infected HL-60 cells, the infected HL-60 cells were plated onto 8-well culture chamber slide and allowed to adhere for 1 h in the 37°C incubator. The cells were fixed with 4% formaldehyde for 10 min at room temperature and permeabilized with 0.1% TX-100 in PBS for 10 min. Subsequently, the cells were stained with rabbit Rab 5 antibody (1:1,000; Cell Signaling Technology) overnight at 4°C. The antigen slides were washed with PBS to remove unbound primary antibody and incubated with second antibody (Alexa Fluor 488-conjugated goat anti-rabbit antibody) for 1 h. The slides were washed 3 times with PBS and mounted with the mounting medium containing DAPI. The slides were then examined with a Zeiss LSM 700 laser scanning confocal microscope (Carl Zeiss Optronics GmbH, Oberkochen, Germany).

### Preparation of axenic media.

The axenic media were prepared essentially as described previously as per the compositions and concentrations outlined by Omsland et al. (21). As per the experimental variability, the medium contained glucose 6-phosphate (G6P), ATP, or both. Similarly, the pH of the media was modified as per the experimental need.

### Protein synthesis by [^35^S]Cys-Met incorporation.

Protein synthesis in cell-free purified phagosome fractions containing *E. chaffeensis* or A. phagocytophilum was measured by incorporation of [^35^S]Cys-Met (PerkinElmer, Waltham, MA) as we described previously (20). Microcentrifuge tubes containing purified phagosome fractions in 500 μl of axenic medium supplemented with 70 μCi of [^35^S]Cys-Met were incubated at 37°C for 24 h in a tri-gas humidified incubator set to maintain 2.5% O_2_. The phagosomes containing rickettsial organisms were pelleted at the end of incubation by centrifugation at 15,000 × *g* for 15 min at 4°C, washed with K-36 buffer (0.05 M K_2_HPO_4_, 0.05 M KH_2_PO_4_, 0.1 M KCl, 0.15 M NaCl, at pH 7.0) twice, and disrupted by adding 30 μl of 2× SDS-PAGE sample buffer and by boiling for 5 min (20). Ten microliters of lysate each was then transferred to a tube containing 5 ml of Biosafe liquid II and used for quantification of [^35^S]Cys-Met incorporation using the protocol 4 (^35^S) in a liquid scintillation counter (Tri-Carb 2100TR; PerkinElmer). For visualization of the radiolabel incorporation into bacterial proteins, equal volumes of sample lysates were resolved by SDS-PAGE, and the gel was dried and exposed to an X-ray film. Similarly, cell-free growth experiments were carried out in the absence of [^35^S]Cys-Met, resolved on an SDS-PAGE gel, and stained using a silver staining kit (Thermo Fisher Scientific, Waltham, MA) as per the manufacturer’s recommendations. To estimate the protein concentration, cell suspensions were concentrated and lysed in 1% SDS solution for 5 min at 100°C, and the total protein concentration was determined using a protein assay kit (Bio-Rad, Hercules, CA).

### DNA synthesis by [^3^H]thymidine incorporation.

Purified A. phagocytophilum- or *E. chaffeensis*-containing phagosomes were also assessed for incorporation of [^3^H]thymidine (PerkinElmer, Waltham, MA) to measure bacterial DNA synthesis (30). Briefly, rickettsia-containing phagosomes were incubated for 24 h at 37°C with 2.5% O_2_ in microcentrifuge tubes containing 500 μl of medium supplemented with 20 μCi of [^3^H]thymidine and 70 μCi of [^35^S]Cys-Met. Rickettsial phagosomes were pelleted at 15,000 × *g* for 15 min at 4°C, washed with K-36 twice, lysed in 30 μl of 2× SDS-PAGE sample buffer, and then boiled for 5 min. Ten microliters of lysate each was added into 5 ml of Biosafe liquid II (Grainger, Hartford, CT) and used for quantification of [^3^H]thymidine incorporation (protocol 10, ^3^H) and [^35^S]Cys-Met incorporation (protocol 4, ^35^S) using a liquid scintillation counting machine (Tri-Carb 2100TR; PerkinElmer), respectively.

### RNA synthesis assessed by real-time qRT-PCR.

Real-time qRT-PCR was performed to measure A. phagocytophilum or *E. chaffeensis* 16S rRNA. In brief, cultures of A. phagocytophilum or *E. chaffeensis* cells grown in several T150 flasks were used in recovering cell-free phagosomes. Triplicate samples of rickettsia-containing phagosomes were incubated for 0, 2, 6, 12, and 24 h with 500 μl of axenic medium at pH 7.0 containing G6P and ATP at 37°C with 2.5% O_2_. At the end of each incubation time, phagosomes were recovered by centrifugation at 15,000 × *g* for 10 min at 4°C. The pellets were then resuspended in TRI reagent solution and used to isolate total RNA as per the TRI reagent protocol (Sigma-Aldrich, St. Louis, MO). The final recovered RNA from each tube was resuspended in 25 μl of nuclease-free water and treated with RQ1 DNase (Thermo Fisher Scientific, Waltham, MA) to remove residual genomic DNAs. RNA from each tube was diluted 1:1,000 in nuclease-free water, and 2 μl each was used in a 25-μl reaction mixture in performing TaqMan probe-based real-time RT-PCR targeted to A. phagocytophilum or *E. chaffeensis* 16S RNA as previously described (31). RNA levels in each sample were expressed by threshold cycle (*C_T_*) values. Variation among triplicates for each time point was calculated and is presented with the respective standard deviations (SD) observed. Fold changes were calculated relative to *C_T_* values observed for RNA recovered before incubation (0 h) compared to different incubation times. The data were then assessed for statistical significance.

### Sodium dodecyl sulfate-polyacrylamide gel electrophoresis.

SDA-PAGE analysis was performed as previously described (20). Briefly, 5 μl of NuPAGE SDS sample buffer and 2 μl of NuPAGE reducing agent (Invitrogen/Thermo Fisher Scientific, Waltham, MA) were added to 10 μl of rickettsial phagosomes after cell-free incubation experiments in the axenic medium and then incubated at 100°C for 5 min, transferred to Mini-PROTEAN precast Bis-Tris 4 to 14% polyacrylamide gels (Bio-Rad, Hercules, CA), and subjected to electrophoresis (100 mA/gel for 60 min). The gels were stained using a silver staining kit (Thermo Fisher Scientific) according to the manufacturer’s recommendations.

### Western blot analysis to assess protein synthesis.

For detection of the DnaK and P28-OMP19 in *E. chaffeensis* or the DnaK in A. phagocytophilum, the electrophoresed proteins described above were transferred onto a nitrocellulose membrane (Thermo Fisher Scientific, Waltham, MA) by electro-blotting using an electrophoretic transfer unit (Bio-Rad, Hercules, CA). Protein transfer buffer was prepared as per the manufacturer’s instructions and used in the protein transfer protocols. Subsequently, expression of *E. chaffeensis* DnaK and P28-OMP19 was assessed using the polyclonal rabbit antisera raised against a recombinant *E. chaffeensis* proteins for DnaK and monoclonal antibody against P28-OMP19 prepared in the murine host, respectively (32). The secondary anti-rabbit or anti-mouse antibody conjugated with horseradish peroxidase (Sigma-Aldrich, St. Louis, MO) and Super Signal West Pico chemiluminescent substrate (Thermo Fisher Scientific, Waltham, MA) were used for signal detection, respectively.

### Statistical analysis.

Differences in protein synthesis, DNA synthesis, and RNA expression between groups were examined using Student's *t* test with online software (http://www.socscistatistics.com/tests/studentttest/Default.aspx), with *P* < 0.05 considered significant.

## References

[1] Ismail N, Bloch KC, McBride JW. 2010. Human ehrlichiosis and anaplasmosis. Clin Lab Med 30:261–292. doi:10.1016/j.cll.2009.10.004. 20513551 PMC2882064

[2] Lambert JS. 2020. An overview of tickborne infections in pregnancy and outcomes in the newborn: the need for prospective studies. Front Med (Lausanne) 7:72. doi:10.3389/fmed.2020.00072. 32211414 PMC7069275

[3] Brett ME, Hinckley AF, Zielinski-Gutierrez EC, Mead PS. 2014. U.S. healthcare providers' experience with Lyme and other tick-borne diseases. Ticks Tick Borne Dis 5:404–408. doi:10.1016/j.ttbdis.2014.01.008. 24713280 PMC4625905

[4] Leiby DA, Gill JE. 2004. Transfusion-transmitted tick-borne infections: a cornucopia of threats. Transfus Med Rev 18:293–306. doi:10.1016/j.tmrv.2004.07.001. 15497129

[5] Reine NJ. 2004. Infection and blood transfusion: a guide to donor screening. Clin Tech Small Anim Pract 19:68–74. doi:10.1053/j.ctsap.2004.01.002. 15179926 PMC7129287

[6] Mascarenhas TR, Silibovsky RS, Singh P, Belden KA. 2018. Tick-borne illness after transplantation: case and review. Transpl Infect Dis 20:e12830. doi:10.1111/tid.12830. 29277955

[7] Chen SM, Dumler JS, Bakken JS, Walker DH. 1994. Identification of a granulocytotropic *Ehrlichia* species as the etiologic agent of human disease. J Clin Microbiol 32:589–595. doi:10.1128/JCM.32.3.589-595.1994. 8195363 PMC263091

[8] Thomas RJ, Dumler JS, Carlyon JA. 2009. Current management of human granulocytic anaplasmosis, human monocytic ehrlichiosis and *Ehrlichia ewingii* ehrlichiosis. Expert Rev Anti Infect Ther 7:709–722. doi:10.1586/eri.09.44. 19681699 PMC2739015

[9] Zhang J-Z, Sinha M, Luxon BA, Yu X-J. 2004. Survival strategy of obligately intracellular *Ehrlichia chaffeensis*: novel modulation of immune response and host cell cycles. Infect Immun 72:498–507. doi:10.1128/iai.72.1.498-507.2004. 14688131 PMC350901

[10] Rikihisa Y. 1999. Clinical and biological aspects of infection caused by *Ehrlichia chaffeensis*. Microbes Infect 1:367–376. doi:10.1016/s1286-4579(99)80053-7. 10602669

[11] Zhang J-Z, Popov VL, Gao S, Walker DH, Yu X-J. 2007. The developmental cycle of *Ehrlichia chaffeensis* in vertebrate cells. Cell Microbiol 9:610–618. doi:10.1111/j.1462-5822.2006.00812.x. 16987329

[12] Troese MJ, Carlyon JA. 2009. *Anaplasma phagocytophilum* dense-cored organisms mediate cellular adherence through recognition of human p-selectin glycoprotein ligand 1. Infect Immun 77:4018–4027. doi:10.1128/IAI.00527-09. 19596771 PMC2738047

[13] Omsland A. 2012. Axenic growth of *Coxiella burnetii*. Adv Exp Med Biol 984:215–229. doi:10.1007/978-94-007-4315-1_11. 22711634

[14] Omsland A, Beare PA, Hill J, Cockrell DC, Howe D, Hansen B, Samuel JE, Heinzen RA. 2011. Isolation from animal tissue and genetic transformation of *Coxiella burnetii* are facilitated by an improved axenic growth medium. Appl Environ Microbiol 77:3720–3725. doi:10.1128/AEM.02826-10. 21478315 PMC3127619

[15] Vu CHT, Lee HG, Chang YK, Oh HM. 2018. Axenic cultures for microalgal biotechnology: establishment, assessment, maintenance, and applications. Biotechnol Adv 36:380–396. doi:10.1016/j.biotechadv.2017.12.018. 29292155

[16] Sandoz KM, Beare PA, Cockrell DC, Heinzen RA. 2016. Complementation of arginine auxotrophy for genetic transformation of *Coxiella burnetii* by use of a defined axenic medium. Appl Environ Microbiol 82:3042–3051. doi:10.1128/AEM.00261-16. 26969695 PMC4959063

[17] Bitew MA, Khoo CA, Neha N, De Souza DP, Tull D, Wawegama NK, Newton HJ, Sansom FM. 2018. De novo NAD synthesis is required for intracellular replication of *Coxiella burnetii*, the causative agent of the neglected zoonotic disease Q fever. J Biol Chem 293:18636–18645. doi:10.1074/jbc.RA118.005190. 30315113 PMC6290155

[18] Beare PA, Jeffrey BM, Long CM, Martens CM, Heinzen RA. 2018. Genetic mechanisms of *Coxiella burnetii* lipopolysaccharide phase variation. PLoS Pathog 14:e1006922. doi:10.1371/journal.ppat.1006922. 29481553 PMC5843353

[19] Omsland A, Sager J, Nair V, Sturdevant DE, Hackstadt T. 2012. Developmental stage-specific metabolic and transcriptional activity of *Chlamydia trachomatis* in an axenic medium. Proc Natl Acad Sci U S A 109:19781–19785. doi:10.1073/pnas.1212831109. 23129646 PMC3511728

[20] Eedunuri VK, Zhang Y, Cheng C, Chen L, Liu H, Omsland A, Boyle D, Ganta RR. 2018. Protein and DNA synthesis demonstrated in cell-free *Ehrlichia chaffeensis* organisms in axenic medium. Sci Rep 8:9293. doi:10.1038/s41598-018-27574-z. 29915240 PMC6006305

[21] Omsland A, Cockrell DC, Howe D, Fischer ER, Virtaneva K, Sturdevant DE, Porcella SF, Heinzen RA. 2009. Host cell-free growth of the Q fever bacterium *Coxiella burnetii*. Proc Natl Acad Sci U S A 106:4430–4434. doi:10.1073/pnas.0812074106. 19246385 PMC2657411

[22] Omsland A, Hackstadt T, Heinzen RA. 2013. Bringing culture to the uncultured: *Coxiella burnetii* and lessons for obligate intracellular bacterial pathogens. PLoS Pathog 9:e1003540. doi:10.1371/journal.ppat.1003540. 24039571 PMC3764191

[23] Dumler JS, Barbet AF, Bekker CP, Dasch GA, Palmer GH, Ray SC, Rikihisa Y, Rurangirwa FR. 2001. Reorganization of genera in the families Rickettsiaceae and Anaplasmataceae in the order Rickettsiales: unification of some species of Ehrlichia with Anaplasma, Cowdria with Ehrlichia and Ehrlichia with Neorickettsia, descriptions of six new species combinations and designation of Ehrlichia equi and ‘HGE agent’ as subjective synonyms of Ehrlichia phagocytophila. Int J Syst Evol Microbiol 51:2145–2165. doi:10.1099/00207713-51-6-2145. 11760958

[24] Pritt BS, Allerdice MEJ, Sloan LM, Paddock CD, Munderloh UG, Rikihisa Y, Tajima T, Paskewitz SM, Neitzel DF, Hoang Johnson DK, Schiffman E, Davis JP, Goldsmith CS, Nelson CM, Karpathy SE. 2017. Proposal to reclassify *Ehrlichia muris* as *Ehrlichia muris subsp. muris subsp. nov*. and description of *Ehrlichia muris subsp. eauclairensis subsp. nov.,* a newly recognized tick-borne pathogen of humans. Int J Syst Evol Microbiol 67:2121–2126. doi:10.1099/ijsem.0.001896. 28699575 PMC5775894

[25] Popov VL, Chen SM, Feng HM, Walker DH. 1995. Ultrastructural variation of cultured *Ehrlichia chaffeensis*. J Med Microbiol 43:411–421. doi:10.1099/00222615-43-6-411. 7473674

[26] Dedonder SE, Cheng C, Willard LH, Boyle DL, Ganta RR. 2012. Transmission electron microscopy reveals distinct macrophage- and tick cell-specific morphological stages of *Ehrlichia chaffeensis*. PLoS One 7:e36749. doi:10.1371/journal.pone.0036749. 22615806 PMC3352939

[27] Bruckert WM, Abu Kwaik Y. 2015. Complete and ubiquitinated proteome of the Legionella-containing vacuole within human macrophages. J Proteome Res 14:236–248. doi:10.1021/pr500765x. 25369898 PMC4286187

[28] Aeberhard L, Banhart S, Fischer M, Jehmlich N, Rose L, Koch S, Laue M, Renard BY, Schmidt F, Heuer D. 2015. The proteome of the isolated *Chlamydia trachomatis* containing vacuole reveals a complex trafficking platform enriched for retromer components. PLoS Pathog 11:e1004883. doi:10.1371/journal.ppat.1004883. 26042774 PMC4456400

[29] Urwyler S, Nyfeler Y, Ragaz C, Lee H, Mueller LN, Aebersold R, Hilbi H. 2009. Proteome analysis of *Legionella* vacuoles purified by magnetic immunoseparation reveals secretory and endosomal GTPases. Traffic 10:76–87. doi:10.1111/j.1600-0854.2008.00851.x. 18980612

[30] Kaplan LA, Bott TL, Bielicki JK. 1992. Assessment of [^3^H]thymidine incorporation into DNA as a method to determine bacterial productivity in stream bed sediments. Appl Environ Microbiol 58:3614–3621. doi:10.1128/AEM.58.11.3614-3621.1992. 16348806 PMC183152

[31] Sirigireddy KR, Ganta RR. 2005. Multiplex detection of *Ehrlichia* and *Anaplasma* species pathogens in peripheral blood by real-time reverse transcriptase-polymerase chain reaction. J Mol Diagn 7:308–316. doi:10.1016/S1525-1578(10)60559-4. 15858156 PMC1867522

[32] Zhang T, Kedzierska-Mieszkowska S, Liu H, Cheng C, Ganta RR, Zolkiewski M. 2013. Aggregate-reactivation activity of the molecular chaperone ClpB from *Ehrlichia chaffeensis*. PLoS One 8:e62454. doi:10.1371/journal.pone.0062454. 23667479 PMC3646808

